# Cost-effectiveness of the My Therapy self-directed therapy program for rehabilitation patients: A stepped wedge cluster randomised trial

**DOI:** 10.1177/02692155251347756

**Published:** 2025-06-12

**Authors:** Natasha K Brusco, Sara L Whittaker, Christina L Ekegren, Meg E Morris, Nicholas F Taylor, Annemarie L Lee, Lisa Somerville, Natasha A Lannin, Rania Abdelmotaleb, Libby Callaway, Keith D Hill, The My Therapy Consortium

**Affiliations:** 1 Rehabilitation, 589488 Ageing and Independent Living (RAIL) Research Centre, 2541 Monash University, Frankston, VIC, Australia; 2 Academic and Research Collaborative in Health (ARCH), 2080 La Trobe University, Bundoora, VIC, Australia; 3 Allied Health Clinical Research Office, Eastern Health, Box Hill, VIC, Australia; 4 Department of Physiotherapy, 2541 Monash University, Frankston, VIC, Australia; 5 Victorian Comprehensive Cancer Centre Alliance, Melbourne, VIC, Australia; 6 Department of Neuroscience, 2541 Monash University, 5392 The Alfred Centre, Melbourne, VIC, Australia; 7 Eastern Health, Box Hill, VIC, Australia

**Keywords:** Rehabilitation, self-management, economic evaluation, cost of implementation

## Abstract

**Objective:**

To determine if the My Therapy self-management program could be implemented without increasing the rehabilitation admission cost, from a health service perspective.

**Design:**

Economic evaluation, including a cost-effectiveness analysis.

**Setting:**

Australian rehabilitation wards (*n* = 9).

**Participants:**

Rehabilitation inpatients with any diagnosis.

**Intervention:**

My Therapy: a self-directed therapy program shown to increase daily inpatient rehabilitation participation dosage time by 38%.

**Main Measures:**

Outcomes included cost (rehabilitation admission and all-cause 30-day readmissions), and effect (minimal clinically important difference in functional independence (FIM^TM^), and quality-adjusted life years (EQ-5D-5L)), to estimate incremental cost-effectiveness ratios (ICERs).

**Results:**

There were 2363 participants, with a mean age of 77 (SD 13) years, 62% female, and 27% with cognitive impairment. My Therapy costs $5 (SD $2) per patient/day to implement, excluding opportunity costs. Estimated differences in effect were non-significant for the proportion of participants achieving a minimal clinically important difference in function (control 31%, intervention 36%; OR: 1.08, 95% CI: 0.77, 1.53), and quality-adjusted life years (mean difference −0.01, 95% CI: −0.04 to 0.02). Estimated differences in cost were also non-significant (OR: 1.06, 95% CI: 0.97, 1.16). ICERs were also non-significant. Post hoc, it was determined that the cost/minute of daily therapy participation was $14/minute for control and $11/minute for intervention conditions.

**Conclusions:**

The My Therapy self-management program was implemented without increasing rehabilitation admission and all-cause 30-day readmission costs. However, clinical differences were not detected. There may have been a small reduction in cost/minute for daily therapy participation.

## Introduction

Current models of inpatient rehabilitation provide as little as one hour per day of rehabilitation therapy,^1^ far below clinical guidelines recommendations (three hours per day).^2^ This is important given the positive dose–response relationship between rehabilitation therapy participation and patient outcomes,^1,3–5^ and the high cost of inpatient adult physical rehabilitation services.^6–9^ With respect to the dose of therapy in rehabilitation, a systematic review of additional physiotherapy delivered in a subacute setting, including a mixed rehabilitation cohort showed that additional physiotherapy reduced rehabilitation length of stay while improving function and quality of life.^10^ In addition, it has been shown that increasing multi-disciplinary therapy dose has a positive impact on patient outcomes.^11^ A fully powered randomised controlled trial of additional occupational therapy and physiotherapy rehabilitation therapy delivered over the weekend to a mixed inpatient rehabilitation cohort showed that increasing the dosage of rehabilitation by providing more therapy to rehabilitation patients, increased function, improved quality of life, reduced costs, length of rehabilitation stay and frequency of re-admissions.^11^

To maximise the effectiveness and cost-effectiveness of inpatient rehabilitation, inpatient models of care must explore effective strategies to increase therapy participation. Examples include the provision of additional supervised weekday^5,12^ and weekend rehabilitation sessions,^1,13^ as well as patient self-directed rehabilitation programs (self-management) completed outside of supervised therapy sessions.^1,13–16^ While some of these strategies have strong evidence to support their cost-effectiveness, e.g., additional weekend rehabilitation,^1,15^ the cost-effectiveness of the provision of self-directed therapy programs during inpatient rehabilitation is yet to be explored despite evidence that this can increase rehabilitation dosage.^17^

My Therapy is an example of a self-directed rehabilitation program that can be completed outside of supervised therapy sessions.^16^ Individual My Therapy programs are based on patient goals co-created by the patient, occupational therapist and physiotherapist. Earlier studies^18,19^ and a recent process evaluation conducted alongside the My Therapy stepped wedge randomised controlled trial,^18^ demonstrated that participation in My Therapy increased the dosage of therapy participation by 100‒182 minutes per week.^17,20^

The aim of this economic evaluation was to establish if the My Therapy self-directed rehabilitation program could be implemented without increasing the cost of a rehabilitation admission, and if it was cost-effective for gains in function, and quality of life from a health service perspective.

## Methods

This economic evaluation has been reported in accordance with the Consolidated Health Economic Evaluation Reporting Standards.^19^ The economic analysis plan has been previously published.^21^ Multi-site ethics approval was received from the Alfred Hospital Human Research Ethics Committee on 13 January 2021 (Approval 69610), with site-specific approvals subsequently received from each participating health service (Alfred Hospital, Approval 758/20; Eastern Health, Approval S21-004-69610; Cabrini Health, Approval 11-04-03-21; Healthscope via La Trobe Human Research Ethics Committee, Approval 758/20), and the trial was prospectively registered on the Australian New Zealand Clinical Trials Registry on 22^nd^ March 2021 (ACTRN12621000313831).

The study population included adults aged 18+ years admitted to inpatient rehabilitation for any injury or illness condition that required rehabilitation. An opt-out approach to consent was used. Data were collected over four health services, with nine rehabilitation wards in total, including two home-based wards. A stepped wedge cluster randomised controlled trial design was conducted over nine six-week blocks, with wards under the usual care conditions in Block 1, followed by one ward transitioning to the intervention condition per block, resulting in all wards being under intervention conditions by Block 9.

Details of usual care inpatient rehabilitation and the My Therapy intervention have been described in the trial protocol.^21,22^ In summary, participants under control and intervention conditions received usual care inpatient rehabilitation from the multidisciplinary team, with participants under intervention conditions also receiving the ‘My Therapy’ program. My Therapy is based on the principles of self-management and involves a self-directed rehabilitation program being completed independently by the patient after being prescribed by the treating occupational therapist and physiotherapist.

### Clinical/effectiveness outcomes

Outcomes included the proportion of participants who achieved a minimal clinically important difference in the total Functional Independence Measure (FIM^TM^)^23^ score (22 points) from admission to discharge.^24^ The FIM^TM^ was measured by FIM^TM^-credentialled members of the multi-disciplinary team on admission and discharge to rehabilitation. In addition, the change in utility index from admission to discharge (5-level EQ-5D (EQ-5D-5L)) estimated quality adjusted life years (QALYs).^25^ The EQ-5D-5L raw scores were self-reported by the participant (or a proxy) to the treating therapists or research project site coordinator on admission and discharge to rehabilitation. Conversion of the raw scores into a utility index was via utility weights derived from an Australian population.^25^ While harms were not reported as part of the economic evaluation, they were reported as part of the main clinical analysis.^22^

### Cost of the My Therapy program (economic costs and opportunity costs of the intervention)

To establish the cost of the My Therapy intervention per participant, per day, a cost analysis was completed. This included (a) the cost of My Therapy during the block immediately prior to implementation (implementation preparation) including but not limited to staff education, and the purchase of My Therapy exercise equipment; and (b) the cost of My Therapy following implementation, including economic costs, as well as opportunity costs. Economic costs were costs in addition to usual care, such as the cost of My Therapy printing and My Therapy hand exercise equipment purchased. Opportunity costs were costs absorbed within usual care resources (i.e. additional resources not provided for wards under intervention conditions or preparing to transition to intervention conditions), such as therapist time to attend My Therapy education or time to prescribe the My Therapy programs. The cost analysis included up to nine time points (one time point per block; depending on when the ward transitioned to My Therapy conditions) across eight wards.

Intervention implementation costs were valued at market rates and these included exercise equipment and material purchased for participant use during My Therapy, education of staff and participants, site co-ordinator time (excluding research-only tasks such as data collection), marketing and communication, and therapist time to prescribe and progress My Therapy programs. Health service staff costs were valued as a Grade 2, Year 3 (mid-range) therapist for clinicians, and as a Grade 3, Year 4 (senior) therapist for site coordinators, based on the relevant Enterprise Bargaining Agreement (Supplementary Table 1), in addition to adding organisation oncosts of 25%. Weekend loadings were not applied. All costs were converted into 2021/22 Australian Dollars. There were several assumptions that underpinned this cost analysis (detailed list of assumptions available from authors on request). These included the exclusion of research related costs from analysis; the exclusion of “incidental” medical and nursing time spent encouraging participants to complete My Therapy; and staff costs calculated as “opportunity” costs when considering time spent learning about My Therapy and delivering My Therapy to participants. To calculate the cost of implementation of *My Therapy per day*, the daily amount was calculated by dividing the total cost of implementation for the block by 42 days (*cost of My Therapy implementation per day* *=* *total costs of implementation for each block/42*). The cost of *My Therapy implementation per patient, per day* was calculated by dividing the daily costs of My Therapy implementation by the occupied bed number on one day during the audit period (*cost of implementation of My Therapy per patient, per day* *=* *cost of My Therapy implementation per day/occupied bed number*).

### Cost-effectiveness of the My Therapy program

We calculated incremental cost-effectiveness ratios. Health services costs for the individual participant rehabilitation admission and all cause 30-day re-admissions were valued by each of the four participating health services based on their standard reporting processes. Where available, this was broken down into direct and indirect hospital costs, with allocations for the different clinical services such as medical, nursing, allied health, pathology, and pharmacy. Due to access to the individual participant data, economic modelling and characterising sources of uncertainty were not completed. Cost data were provided in Australian Dollars from the 2020/21 and 2021/22 financial years. Cost data from the 2020/21 financial year was inflated by consumer price index so that all cost data in the analyses were presented as 2021/22 Australian Dollars.^26^

Data transformation included adding the cost of the My Therapy intervention to the cost of the rehabilitation admissions. Based on the cost analysis described in this economic evaluation, the daily economic cost of the My Therapy intervention (i.e. excluding opportunity costs) was established and then added to the health service reported cost. The daily economic cost of My Therapy per participant per admission was calculated by multiplying the daily cost (economic cost only) by the average length of stay for the intervention group.

Cost of admission for rehabilitation participants under the My Therapy intervention condition = Health services costs for the rehabilitation admission + (daily ‘economic’ cost of the My Therapy intervention x Participant rehabilitation length of stay)

Data transformation also included a 60-day sunset clause for the 15 rehabilitation admissions that exceeded a 60-day length of stay (*n* = 15/2363 (0.06%), range 62 to 167 days). This transformation included capping the length of stay and the associated costs to 60 days. This was carried out to minimise the skewed nature of hospital cost data^27^ and to account for the common discharge barriers that are not related to the My Therapy intervention, such as awaiting a residential aged care bed or the appointment of a guardian/power of attorney.^28^

A post-hoc explorative analysis was completed to establish the cost per minute of therapy participation in usual care conditions and in usual care plus My Therapy conditions. The analysis and data for the dosage of rehabilitation participation outcome were completed as part of the clinical trial process evaluation.^20^ Therapy dosage increased by 26 min a day with My Therapy.^20^ The dosage was established using a sub-set of participants from both the usual care and intervention groups.

A health service perspective was chosen for the economic evaluation, as this study aimed to influence health service policy. The time horizon was from rehabilitation admission to rehabilitation discharge, in addition to the 30 days following discharge from rehabilitation (based on all-cause health service re-admissions). No discount rate was applied due to the short time horizon.

### Patient and public involvement

A consumer representative with recent experience in inpatient rehabilitation (VR) was included on the steering committee from the project's outset. They attended quarterly steering committee meetings and responded to ad-hoc requests from investigators throughout the trial. Patient engagement resulted in the inclusion of a nested qualitative study to better understand the patient and carer experience with My Therapy, as well as strategies to better disseminate the results to culturally and linguistically diverse communities.

### Analysis

Cost of implementation: Once the cost of My Therapy implementation was established for each of the wards, this was divided by the number of participant days that were under the intervention conditions, to establish the cost per participant per day, with the mean and standard deviation across the wards reported. A sensitivity analysis was completed, moving the costs of the site co-ordinator's wage (i.e. an additional research personnel) to opportunity costs on the assumption that future scaling of the My Therapy intervention would have the site coordinator roles absorbed within usual duties by ‘clinical champions’ on the ward. For the cost of implementation data, modelling was not performed, and due to the simple nature of this analysis, sources of uncertainty were not characterised. Explorative sub-group analyses for the cost of My Therapy were completed for public versus private, and hospital-based versus home-based wards.

Cost and effect analysis: Primary cost-effectiveness analyses only included participants admitted for rehabilitation and not those admitted for geriatric evaluation and management. First, the cost per participant was determined for the control and intervention conditions to report if My Therapy could be implemented without an associated increase in inpatient admission cost. Then, incremental cost-effectiveness ratios were calculated for (a) the cost per minimal clinically important difference in FIM™ achieved; and (b) the cost per quality-adjusted life year gained (based on utility index change scores), using the cost as the numerator, and the utility, effectiveness data as the denominator. Costs included rehabilitation length of stay (including acute transfers during rehabilitation), 30-day all-cause hospital re-admissions, as well as the cost of the My Therapy intervention for participants under the intervention conditions. The mean cost difference between the two groups was estimated using a mixed linear regression model with random effects for wards, and fixed effects for block, intervention, and admission FIM score.^29^ The incremental cost-effectiveness ratios were generated through cluster-level bootstrapping of the estimator for both the numerator (estimate of the effect of the intervention on total cost) and the denominator (estimate of the effect of the intervention on either FIM™ minimal clinically important difference being achieved (binary outcome) expressed as an odds ratio, or on EQ-5D-5L utility index change score. A total of 4000 bootstrap replications were performed and then a percentile bootstrap confidence interval was generated from the set of the 4000 estimates of each of these two ratios to get a confidence interval for each of the two incremental cost-effectiveness ratios. The change in utility index from admission to discharge was used to estimate QALYs. Incremental cost-effectiveness ratios were used to determine the probability that My Therapy was less costly and more beneficial compared with usual care alone, using a $AUD50,000 per quality-adjusted life year gained threshold to determine cost-effectiveness.^30,31^ Sub-group analyses were not conducted for the incremental cost-effectiveness ratios.

### Contextual information that may influence findings

The key contextual factor that may have influenced the findings of this study was the COVID-19 pandemic, as this globally and locally affected inpatient rehabilitation service delivery and shifted patient admission demographics.^32,33^ Due to the stepped-wedge design, there were more participants under the intervention conditions in the second half of the data collection period when Victoria, Australia, experienced its most severe state government mandated lockdowns, as well as staff shortages and cancellation of elective surgery.^34^ Three of the key impacts from the COVID-19 pandemic on the current study were (a) hospital visitor restrictions throughout the clinical trial (an important enabler of the My Therapy intervention),^35^ (b) closure of one participating sub-acute rehabilitation ward due to conversion to an acute COVID-ward, noting this ward was replaced by another rehabilitation ward at the same health service, as well as (c) an observed shift in patient admission demographics during the second half of the trial (when most wards were under My Therapy intervention conditions) to include more medical patients (e.g. following a stroke), fewer elective orthopaedic patients, and patients with an overall lower function and quality of life on admission to rehabilitation.^22^

## Results

### Cost of the My Therapy program

Including both economic (My Therapy costs above and beyond usual care costs) and opportunity (My Therapy costs absorbed within usual duties/usual hours) costs, the total cost of the My Therapy intervention was $AUD911,240, over 54 weeks, across 928 participants (based on average occupancy in any 6-week period during intervention conditions). The per participant, per day, cost of the My Therapy intervention (economic and opportunity costs) was $AUD26 (SD $AUD21) (Supplementary Table 2). When considering economic costs only, the per participant, per day cost was $AUD5 (SD $AUD2) (Supplementary Table 2). The number and cost of the My Therapy units included in the cost analysis are presented in Supplementary Tables 3 and 4 respectively. In the sub-group analysis, there were cost differences between private and public wards with higher costs shown for implementation for patients under public hospitals, as well as between hospital and home-based wards with higher costs shown for implementation for patients receiving home-based rehabilitation (Supplementary Table 5). In the sensitivity analysis, when the cost of the site co-ordinators’ wages were moved from economic to opportunity costs, on the assumption that future scaling of the My Therapy intervention would have the site coordinator roles absorbed within usual duties by ‘clinical champions’ on the ward, the per participant, per day economic cost of the My Therapy intervention was reduced to $AUD0.06 (SD $AUD0.24) (Supplementary Table 2).

### Cost-effectiveness of the My Therapy program

The cost-effectiveness analysis included 2363 participants (control *n* = 1,328, intervention *n* = 1035) with complete cost and effect data, with participant characteristics described in detail in the separate clinical paper.^22^ While the participant data sets are similar between the full clinical evaluation reported separately (*n* = 2550)^22^ and this economic evaluation (*n* = 2363), participant numbers vary slightly as there was a small set of participants who did not have available cost data from the health service and were subsequently removed from the economic evaluation (*n* = 187/2550; 7.3%). In summary, participants on average were 77 (SD = 13) years of age on admission to rehabilitation, 62% were female, 43% were living alone prior to their rehabilitation admission, and the primary reasons for admission to rehabilitation included conditions relating to orthopaedic joint replacements or other orthopaedic conditions (32%), reconditioning following a medical illness or surgery (26%), and stroke (9%). Most participants did not have cognitive impairment (73%), while 22% had mild, and 5% had severe cognitive impairment.

After adjusting for confounders, clinical differences between groups were small with wide confidence intervals for the odds of achieving a minimal clinically important difference in function (Odds Ratio [95% CI] = 1.08 [0.77 to 1.53]), and the difference in quality-adjusted life years gained (Mean difference [95% CI] = −0.01, [−0.04 to 0.02]) (Table 1). Based on the rehabilitation length of stay for the intervention group (15.2 days [SD = 9.7]), and the average daily economic cost of the My Therapy intervention per participant ($5 [SD = $2]), the cost of My Therapy per participant, per admission was $76 (SD = $49). While the intervention group had a longer length of stay for the rehabilitation admission combined with all cause 30-day readmissions (OR [95% CI] = 1.10 [1.02 to 1.37]), there was no increase in costs (OR [95% CI] = 1.06 [0.97 to 1.16]) (Table 2).

**Table 1.** Primary effectiveness outcomes: function (FIM^TM^) and quality of life (EQ-5D-5L).

	Control condition (*n* = 1328)	Intervention condition (*n* = 1025)	Odds ratio/mean difference (95% CI)	*p*	ICC
*FIM^TM^*			*Odds ratio (95% CI)*		
Change in total score, *M* (SD)	16.1 (16.3)	16.8 (17.7)	0.29 (−2.15; 2.74)	.81	0.07
			*Mean difference (95% CI)*		
MCID of FIM^TM^ Achieved, *n (%)*	399 (31%)	369 (36%)	1.08 (0.77; 1.53)	.64	0.10
EQ-5D-5L	(*n* = 1229)	(*n* = 874)	Mean difference (95% CI)		
Change in index score, *M* (SD)	0.17 (0.26)	0.18 (0.29)	−0.009 (−0.04; 0.02)	.55	0.09

FIM = Functional independence measure; MCID = Minimal clinically important difference; 95% CI = 95% Confidence interval; ICC = intracluster correlation coefficient.

**Table 2.** Economic outcomes.

	Control condition *M* (SD) (*n* = 1328)	Intervention condition *M* (SD) (*n* = 1025)	Ratio of geometric means (95% CI)	*p*	*ICC*
Length of stay (days) rehabilitation	13.1 (6.2)	15.2 (9.7)	1.09 (1.01; 1.17)	.02	0.09
Number of 30-day readmission(s)	0.3 (0.9)	0.5 (1.3)	1.07 (0.83; 1.37)	.60	
Length of stay (days) - rehabilitation and 30-day readmission(s)	15.8 (12.9)	19.4 (19.8)	1.10 (1.02; 1.19)	.02	0.04
Cost of rehabilitation admission	$12,851.64 ($12,285.81)	$15,720.13 ($14,792.08)	1.03 (0.95; 1.11)	.41	0.21
Cost of rehabilitation admission and My Therapy (*included for intervention conditions only*)	$12,851.64 ($12,285.81)	$15,795.93 ($14,831.48)	1.04 (0.96; 1.13)	.33	0.21
Cost of rehabilitation admission, My Therapy (*included for intervention conditions only*) and 30-day readmission(s)	$15,867.47 ($19,501.49)	$20,913.50 ($26,909.29)	1.06 (0.97; 1.16)	.21	0.17

All costs are presented as $AUD 2021/2022. MD = Mean difference; OR = odds ratio; 95% CI = 95% Confidence Interval; ICC = intracluster correlation coefficient.

Effect and quality-adjusted life year estimated incremental cost-effectiveness ratio values had very wide confidence intervals that were centred close to $0, so the estimates of the corresponding incremental cost-effectiveness ratio were highly variable. Descriptively, the incremental cost-effectiveness ratio for the proportion of participants who achieved a minimal clinically important difference in function was $1602 (95% CI [−$3925 to 4869]), and the incremental cost-effectiveness ratio per quality-of-life year gained was $78,272 (95% CI [−$1,554,639 to $1,254,316]), noting that the wide confidence intervals represent the high degree of variability in the cost and effect outcomes. The incremental cost difference between the intervention and control conditions was $2955. The cost per quality-of-life year gained for the My Therapy intervention group exceeded the a priori $50,000 willingness to pay threshold.

The results of the post-hoc explorative analysis showed the cost per day of rehabilitation under usual care conditions was $981.04 including 69 min of rehabilitation participation. The cost of rehabilitation under usual care plus My Therapy conditions was $1039.21 and this included 95 min of rehabilitation participation (69 min of supervised therapy and 26 min of My Therapy participation). Under usual care conditions, the cost of therapy participation per minute was $14.22, and under usual care plus My Therapy the cost of therapy participation per minute was $10.94 (23% reduction in cost per minute).

## Discussion

This economic evaluation established that My Therapy could be implemented across multiple health services, without an associated increase in the cost per admission. My Therapy increased therapy participation by 26 min a day and may have reduced the overall cost per minute of therapy participation. However, the addition of My Therapy did not lead to clinically significant improvements in participant function or quality of life compared to usual rehabilitation alone.

The strengths of this economic evaluation include large participant numbers and a prospective randomised design across four health services. A limitation of this economic evaluation was conducting the clinical trial during the height of the COVID-19 pandemic, which influenced the traditional rehabilitation landscape with the presence of hospital visitor restrictions (a key enabler of My Therapy),^35^ the shift in patient admission demographics during the second half of the trial to include more medical and fewer elective orthopaedic patients, and patients with an overall lower function and quality of life on admission to rehabilitation.^22^ As reported in the clinical trial results, participants under My Therapy conditions experienced a longer rehabilitation and 30-day readmission length of stay than control participants and it is likely that this was due to the imbalance in participant characteristics between control and intervention conditions associated with a case-mix change during the COVID-19 pandemic, for example less elective orthopaedic surgery and more complex neurological presentations under the My Therapy intervention conditions.^17^ It is also possible that the dosage of self-management via My Therapy was not adequate for achieving functional gains. Two small trials of self-management in rehabilitation also did not show improvement in function compared to routine care, the authors of these studies also suggested that self-management dosage may have been inadequate.^36,37^ To minimise the data collection burden, a limitation of this economic evaluation is the short time horizon (30 days) that was applied. We recognise that with a shortened time horizon the potential long-term cost savings of increasing rehabilitation therapy may be missed as they would be seen beyond 30 days, including reduced healthcare utilisation (e.g. reduction in presentations to emergency departments, reduction in primary care service use). Future research to include a time horizon of 1 year may be of benefit to observe the clinical and economic benefits of increased rehabilitation therapy. Furthermore, this would allow for additional analysis of quality-adjusted life year data over a longer period of time to determine the accumulation of increase in quality-adjusted life years in the intervention group. Future research may also consider modelling economic outcomes of My Therapy over a longer time horizon, using existing literature for longer-term outcomes of rehabilitation patients with and without higher intensity of rehabilitation therapy group.

A recent systematic review estimated that a self-management program, alongside usual care at a health care service, cost an additional $90 (AUD 2022, 95% CI [−$130 to $310]) per episode of care.^38^ When we excluded costs absorbed within usual care resources in the current study, the My Therapy self-management program, alongside usual care inpatient rehabilitation, came at a comparable cost of $76 (SD$49) per admission. In the context of the full cost of an average 13 day usual care rehabilitation admission, i.e., $AUD13,000 as reported for the current study, the addition of $76 is negligible, especially when results from the related My Therapy process evaluation reported that usual care rehabilitation participation was 69 min per day, and that My Therapy led to participation in an additional 26 min per day, in addition to other patient reported benefits such as improved feelings of empowerment and engagement.^20,22,39^

Based on previous evidence that participating in additional rehabilitation therapy can reduce length of stay, and increase gains in function and quality of life,^10^ the findings from this economic evaluation were unexpected. Possible explanations for the lack of between-group difference include (a) the impact of the COVID-19 pandemic on healthcare delivery (as outlined above, e.g., lack of hospital visitors which was a key facilitator of My Therapy), or (b) that the overall dosage of rehabilitation was insufficient to achieve function-based patient gains.^5,10^ Regarding the dosage, it may be that the increase in therapy participation, i.e., 26 min per day, did not reach the threshold of additional therapy participation, or the recommended total of three hours per day, to impact patient outcomes.^2,5^ This indicates that My Therapy may need to be combined with other rehabilitation strategies aimed at improving dosage so as to reach the minimal effective dosage threshold. In the context of general rehabilitation, other studies have also found that therapy participation in rehabilitation^40^ currently falls short of the recommended three hours per day,^2^ and in the context of stroke rehabilitation, an increase of 100 min per day in therapy participation was required to improve upper limb function.^5^

There are several unanswered questions that may guide future research. For example, what is the social return on investment for My Therapy? The non-monetary value of the My Therapy intervention has been demonstrated in other contexts, including a half-hour increase to the daily dosage of rehabilitation participation,^20^ being feasible with patients with cognitive impairment,^41^ empowering patients during their rehabilitation journey,^35^ and providing families with a meaningful way to support their loved ones in rehabilitation.^35^ However, these benefits were not valued or considered in the current economic evaluation where only health service costs, function and quality of life, were considered. The statistical analysis for costs in this study is presented as geometric means, which were deemed to be most appropriate for the dataset given the diagnostics for the linear fit. However, for some, this could be considered a limitation in the analysis approach.^42^

While clinical differences were not detected in this study, My Therapy increased therapy participation across multiple health services, without an associated increase in overall cost per admission. This study demonstrated a reduction in cost/minute of therapy participation. The narrow health service perspective meant that other possible patient, health service and economic benefits of My Therapy during inpatient rehabilitation were not considered in this economic evaluation.

**Clinical messages**

- My Therapy self-management cost $5 per patient per day, excluding opportunity costs, and did not increase overall rehabilitation admission costs.
- My Therapy is a low-cost intervention that can improve therapy participation during rehabilitation.
- It remains uncertain whether the increased participation in rehabilitation with My Therapy results in improved clinical outcomes.

## Supplemental Material

sj-docx-1-cre-10.1177_02692155251347756 - Supplemental material for Cost-effectiveness of the My Therapy self-directed therapy program for rehabilitation patients: A stepped wedge cluster randomised trial Supplemental material, sj-docx-1-cre-10.1177_02692155251347756 for Cost-effectiveness of the My Therapy self-directed therapy program for rehabilitation patients: A stepped wedge cluster randomised trial by Natasha K Brusco, Sara L Whittaker and Christina L Ekegren, Meg E Morris, Nicholas F Taylor, Annemarie L Lee, Lisa Somerville, Natasha A Lannin, Rania Abdelmotaleb, Libby Callaway, Keith D Hill, in Clinical Rehabilitation

sj-docx-2-cre-10.1177_02692155251347756 - Supplemental material for Cost-effectiveness of the My Therapy self-directed therapy program for rehabilitation patients: A stepped wedge cluster randomised trial Supplemental material, sj-docx-2-cre-10.1177_02692155251347756 for Cost-effectiveness of the My Therapy self-directed therapy program for rehabilitation patients: A stepped wedge cluster randomised trial by Natasha K Brusco, Sara L Whittaker and Christina L Ekegren, Meg E Morris, Nicholas F Taylor, Annemarie L Lee, Lisa Somerville, Natasha A Lannin, Rania Abdelmotaleb, Libby Callaway, Keith D Hill, in Clinical Rehabilitation
