# Supplementary material for: Cost-effectiveness of the My Therapy self-directed therapy program for rehabilitation patients: A stepped wedge cluster randomised trial
Source: Clin Rehabil. 2025 Jun 12;39(7):890–901. doi: 10.1177/02692155251347756 (PMC12198461; doi:10.1177/02692155251347756)
Supplement: sj-docx-1-cre-10.1177_02692155251347756 - Supplemental material for Cost-effectiveness of the My Therapy self-directed therapy program for rehabilitation patients: A stepped wedge cluster randomised trial [file sj-docx-1-cre-10.1177_02692155251347756.docx]

**Cost-effectiveness of a self-directed therapy program (‘My Therapy’) for rehabilitation patients: a pragmatic stepped wedge cluster randomised trial**

**Supplementary material**

Table of Contents

[Supplementary Table 1: Enterprise Bargaining Agreement (EBA) wage references (occupational therapist, physiotherapist) 2](#_Toc194144036)

[Supplementary Table 2: Cost analysis, average cost per participant, per day (including sensitivity analysis) 3](#_Toc194144037)

[Supplementary Table 3: Cost analysis, units utilised across the wards 4](#_Toc194144038)

[Supplementary Table 4: Cost analysis, average cost of units utilised (per participant, per day) 5](#_Toc194144039)

[Supplementary Table 5: Cost analysis, sub-group analysis 6](#_Toc194144040)

# **Supplementary Table 1: Enterprise Bargaining Agreement (EBA) wage references (occupational therapist, physiotherapist)**

| **Professional** | **Grading assumption** | **Weekly salary** | **Hourly rate** | **Organisational oncosts** | **Total hourly costs** |
| --- | --- | --- | --- | --- | --- |
| Occupational therapist | Grade 2, Year 3 | $1815.30 | $47.77 | $11.94 | $59.71 |
| Physiotherapist | Grade 2, Year 3 | $1815.30 | $47.77 | $11.94 | $59.71 |
| Site co-ordinator | Grade 3, Year 4 | $2156.10 | $56.74 | $14.19 | $70.93 |
| *Source:* [*https://www.fwc.gov.au/document-search/view/3/aHR0cHM6Ly9zYXNyY2RhdGFwcmRhdWVhYS5ibG9iLmNvcmUud2luZG93cy5uZXQvZW50ZXJwcmlzZWFncmVlbWVudHMvMjAyMi8xMS9BRTUxODE0Ni5wZGY1?sid=&q=IAN%24%24ALLIED%24%24HEALTH%24%24PROFESSIONALS%24%24VICT%24%24PUBLIC%24%24SECTOR*](https://www.fwc.gov.au/document-search/view/3/aHR0cHM6Ly9zYXNyY2RhdGFwcmRhdWVhYS5ibG9iLmNvcmUud2luZG93cy5uZXQvZW50ZXJwcmlzZWFncmVlbWVudHMvMjAyMi8xMS9BRTUxODE0Ni5wZGY1?sid=&q=IAN%24%24ALLIED%24%24HEALTH%24%24PROFESSIONALS%24%24VICT%24%24PUBLIC%24%24SECTOR) | | | | | |
| *All costs presented as $AUD 2021/2022* | | | | | |

# **Supplementary Table 2: Cost analysis, average cost per participant, per day (including sensitivity analysis)**

|  | **TOTAL Cost of My Therapy implementation,** over 54 weeks, across 928 participants  (sum) | **Blocks ward under intervention conditions**  (n) | **Cost of implementation, per block**  mean (SD) | **Cost of implementation, per day**  mean (SD) | **Number of participants under My Therapy conditions on ward** (n= sum, mean per block) | **Cost of implementation per participant, per day**  mean (SD) |
| --- | --- | --- | --- | --- | --- | --- |
| *Economic and opportunity costs* | | | | | | |
| All wards | $911,240 | 36 | $25,312 ($14,611) | $603 ($348) | 928, 26 | $26 ($21) |
| *Economic costs only* | | | | | | |
| All wards | $180,568 | 36 | $5,016 ($2,218) | $119 ($53) | 928, 26 | $5 ($2) |
| *Sensitivity analysis of economic costs assuming site co-ordinator wage absorbed as opportunity costs* | | | | | | |
| All wards | $2,676 | 36 | $74 ($306) | $2 ($7) | 928, 26 | $0.06 ($0.24) |
| *All costs presented as $AUD 2021/2022* | | | | | | |

# **Supplementary Table 3: Cost analysis, units utilised across the wards**

| **Area of resource** | **Total Units**  *(n) (range)* | **Units per ward**  *Mean (SD)* |
| --- | --- | --- |
| *Exercise materials purchased* |  |  |
| Weights (number of instances purchased) | 2 (0-2) | 0.25 (0.46) |
| Written resources (number of instances purchased) | 10 (0-10) | 1.25 (1.75) |
| Other (e.g. cycling pedals, long handled aids, mirrors) (number of instances purchased) | 10 (0-10) | 1.25 (1.39) |
| *Education sessions to staff* |  |  |
| Number of OT/PT education sessions | 90 (0-8) | 11.25 (5.75) |
| Number of OT/PT attendees at sessions | 202 (0-28) | 25.25 (14.15) |
| Time spent completing OT/PT education (minutes) | 3950 (0-360) | 493.75 (257.96) |
| Number of education sessions completed for other staff | 51 (0-7) | 6.38 (3.25) |
| Number of other staff attendees at sessions | 152 (0-30) | 19 (11.71) |
| Time spent completing other staff education (minutes) | 1432 (0-360) | 179 (166.11) |
| Time spent by co-ordinator preparing education sessions (minutes) | 5410 (0-1020) | 676.25 (447.24) |
| *Communication materials* |  |  |
| Time preparing communication material (minutes) | 3180 (0-300) | 397.50 (205.36) |
| Time delivering My Therapy material (minutes) | 2150 (0-300) | 268.75 (225.91) |
| Time on miscellaneous communication tasks (minutes) | 270 (0-180) | 33.75 (66.96) |
| *Marketing materials* |  |  |
| Materials for My Therapy packs (number of instances purchased) | 14 (0-14) | 1.75 (2.45) |
| Miscellaneous marketing items (e.g. magnets, plastic folders, plastic storage containers) (number of instances purchased) | 4 (0-4) | 0.5 (0.76) |
| *Staff time supporting My Therapy (daily amounts)** | | |
| OT time within supervised sessions (minutes) | 3167 (15-360) | 395.81 (373.95) |
| PT time within supervised sessions (minutes) | 4631 (25-450) | 578.88 (538.67) |
| OT time outside of supervised sessions (minutes) | 2472 (25-450) | 308.94 (220.87) |
| PT time outside of supervised sessions (minutes) | 930 (10-60) | 116.25 (70.95) |
| *Footnote: 44 blocks included (36 implementation + 8 blocks pre-implementation). Staff time only captured during implementation blocks, not pre-implementation blocks. Units per ward mean calculated as a mean of the total units divided by 8 wards.* | | |

# **Supplementary Table 4: Cost analysis, average cost of units utilised (per participant, per day)**

| **Resource area** | **Economic costs**  (per participant, per day)  *mean (SD)* | **Opportunity costs**  (per participant, per day)  *mean (SD)* | **Total costs**  (per participant, per day)  *mean (SD)* |  |
| --- | --- | --- | --- | --- |
| *Cost of exercise material* | | | |  |
| All wards | $0.06 ($0.24) | $0.00 | $0.06 ($0.24) |  |
| *Cost of education for OT and PT staff* | | | |  |
| All wards | $0.27 ($0.49) | $0.13 ($0.16) | $0.39 ($0.64) |  |
| *Cost of education for other staff* | | | |  |
| All wards | $0.04 ($0.10) | Not captured | $0.04 ($0.10) |  |
| *Cost of miscellaneous education* | | | |  |
| All wards | $0.16 ($0.31) | $0.00 | $0.16 ($0.31) |  |
| *OT cost for recommending and progressing My Therapy* |  |  |  |  |
| All wards | $0.00 | $11.03 ($11.52) | $11.03 ($11.52) |  |
| *PT cost for recommending and progressing My Therapy* |  |  |  |  |
| All wards | $0.00 | $9.79 ($8.34) | $9.79 ($8.34) |  |
| *Cost of site co-ordinator* | | | |  |
| All wards | $4.24 ($1.75) | $0.34 ($0.61) | $4.58 ($2.06) |  |
| *Cost of marketing and communication* | | | |  |
| All wards | $0.19 ($0.23) | $0.00 | $0.19 ($0.23) |  |
| TOTAL | $4.96 | $21.29 | $26.24 |  |
| *This table includes the pre-implementation block prior to the crossover to My Therapy conditions (44 blocks included (36 implementation + 8 blocks pre-implementation). All costs presented as $AUD 2021/2022* | | | |  |

# **Supplementary Table 5: Cost analysis, sub-group analysis**

|  | **Blocks ward under intervention conditions**  (n) | **Cost of implementation, per block**  mean (SD) | **Cost of implementation, per day**  mean (SD) | **Number of participants under My Therapy conditions on ward**  (n = sum, average) | **Cost of implementation per participant, per day**  mean (SD) |
| --- | --- | --- | --- | --- | --- |
| **All wards (n=8)** |  |  |  |  |  |
| *Period (i)* | 8 | $5,914 ($2,028) | $141 ($48) | 0 | N/A |
| *Period (ii)* | 8 | $26,634 ($17,067) | $634 ($406) | 204, 26 | $29 ($25) |
| *Period (iii)* | 28 | $23,245 ($13,549) | $553 ($323) | 724, 26 | $24 ($19) |
| **Hospital wards (n=6)** |  |  |  |  |  |
| *Period (i)* | 6 | $5,953 ($2,396) | $142 ($57) | 0 | N/A |
| *Period (ii)* | 6 | $17,583 ($3,544) | $419 ($84) | 164, 27 | $16 ($7) |
| *Period (iii)* | 21 | $17,656 ($3,768) | $420 ($90) | 585, 28 | $15 ($4) |
| **Bed-based wards (n=2)** |  |  |  |  |  |
| *Period (i)* | 2 | $5,797 ($210) | $138 ($5) | 0 | N/A |
| *Period (ii)* | 2 | $53,788 ($3,157) | $1281 ($75) | 40, 20 | $66 ($18) |
| *Period (iii)* | 7 | $40,012 ($18,477) | $953 ($440) | 139, 20 | $49 ($23) |
| **Private hospital wards (n=4)** |  |  |  |  |  |
| *Period (i)* | 4 | $5,408 ($1,190) | $129 ($28) | 0 | N/A |
| *Period (ii)* | 4 | $18,395 ($2,333) | $438 ($56) | 107, 27 | $18 ($7) |
| *Period (iii)* | 11 | $16,861 ($1,326) | $401 ($32) | 303, 28 | $15 ($2) |
| **Public hospital wards (n=4)** |  |  |  |  |  |
| *Period (i)* | 4 | $6,419 ($2,738) | $153 ($65) | 0 | N/A |
| *Period (ii)* | 4 | $34,874 ($22,207) | $830 ($529) | 97, 24 | $40 ($32) |
| *Period (iii)* | 17 | $27,375 ($16,203) | $652 ($386) | 421, 25 | $30 ($23) |
| **Public hospital wards (excluding bed-based wards) (n=2)** |  |  |  |  |  |
| *Period (i)* | 2 | $7,042 ($4,572) | $168 ($109) | 0 | N/A |
| *Period (ii)* | 2 | $15,960 ($6,210) | $380 ($148) | 57, 29 | $14 ($8) |
| *Period (iii)* | 10 | $18,530 ($5,289) | $441 ($126) | 282, 28 | $16 ($6) |

*All costs presented as $AUD 2021/2022. Period (i) refers to 6 weeks prior to implementation of My Therapy; Period (ii) refers to the First 6-week My Therapy block; Period (iii) refers to Second and subsequent 6 week My Therapy blocks after trial cross over.*
